# Fluctuations of health states in dementia diseases and their impact on the assessment of health today using the EQ-5D-5L: Protocol of a mixed-methods study

**DOI:** 10.3389/fpubh.2023.1031978

**Published:** 2023-03-16

**Authors:** Niklas Weber, Feng Xie, Thomas Kohlmann, Wiebke Mohr, Moritz Platen, Anika Rädke, Ingo Kilimann, Lidia Engel, Bernhard Michalowsky

**Affiliations:** ^1^Translational Health Care Research, German Center for Neurodegenerative Diseases (DZNE) Site Rostock/Greifswald, Greifswald, Germany; ^2^Department of Health Research Methods, Evidence and Impact, McMaster University, Hamilton, ON, Canada; ^3^Center for Health Economics and Policy Analysis, McMaster University, Hamilton, ON, Canada; ^4^Section Methods of Community Medicine, Institute for Community Medicine, University Medicine Greifswald, Greifswald, Germany; ^5^Clinical Dementia Research, German Center for Neurodegenerative Diseases (DZNE) Site Rostock/Greifswald, Rostock, Germany; ^6^School of Public Health and Preventive Medicine, Monash University, Melbourne, VIC, Australia

**Keywords:** dementia, health state fluctuation, patient reported outcome measures, health related quality of life, Psychometrics, EQ-5D

## Abstract

**Introduction:**

The EQ-5D is a widely used health-related quality of life (HRQoL) instrument. The recall period “today” may miss out on recurrent health fluctuations often observed in people with dementia (PlwD). Thus, this study aims to assess the frequency of health fluctuations, affected HRQoL dimensions and the impact of the health fluctuations on the assessment of health today using the EQ-5D-5L.

**Methods and analysis:**

This mixed-methods study will base on n=50 patient and caregiver dyads and four main study phases: (1) Baseline assessment of patients' socio-demographic and clinical characteristics; (2) caregivers self-completion of a daily diary for 14 days, documenting patient's today's health compared to yesterday, the affected HRQoL dimensions, and events that could have caused the fluctuations; (3) administration of the EQ-5D-5L as self- and proxy-rating at baseline, day seven and day 14; (4) interviewing caregivers on patient's health fluctuation, the consideration of past fluctuations in the assessment of health today using the EQ-5D-5L, and the appropriateness of recall periods to capture health fluctuations on day 14. Qualitative semi-structured interview data will be analyzed thematically. Quantitative analyses will be used to describe the frequency and intensity of health fluctuations, affected dimensions, and the association between health fluctuation and its consideration in the assessment of health today.

**Discussion:**

This study aims to reveal insights into the health fluctuation in dementia, the affected dimensions, and underlying health events, as well as whether individuals adhere to the recall period of health today using the EQ-5D-5L. This study will also provide information about more appropriate recall periods that could better capture health fluctuations.

**Trial registration:**

This study is registered in the German Clinical Trials Register (DRKS00027956).

## Introduction

The EQ-5D-5L is a widely used preference-based generic instrument that measures today's self-rated health-related quality of life (HRQoL) (1). However, the health state can fluctuate from day to day in various chronic diseases such as Alzheimer's and other related dementia diseases (2, 3). People living with dementia (PlwD) and their caregivers often refer to this as “spells of good and bad days” (4). PlwD may have improved overall cognition, function, interest, and initiation on good days, while on bad days, there are frequent verbal repetition, poor memory, increased agitation, and other disruptive behaviors. Thus, clinically important symptom variability appears common in dementia (4). Cognitive and non-cognitive symptoms, such as anxiety, depression, pain, and discomfort can cause changes in the severity of symptoms and affect other health domains, including the patient's daily activities and self-care. Even though the EQ-5D-5L has proven acceptability, feasibility, and validity for moderately cognitively impaired PlwD, health state fluctuation in dementia might still significantly affect the assessment (5–7).

So far, challenges in measuring HRQoL and the impact of health state fluctuations have not been well-understood. Fluctuations of predominantly cognitive symptoms have been assessed clinically for Alzheimer's and Lewy body dementias (8). Sun et al. (2) showed an association of cognitive fluctuations with poorer HRQoL. However, health state fluctuations in HRQoL regarding PlwD has not been assessed yet. Blome et al. (9) showed that fluctuations in HRQoL frequently occur between and within days and impact the assessment of today's health with the EQ-5D-5L in patients with relapsing-remitting and secondary progressive multiple sclerosis. The authors recommended that a longer recall period or a mean of several measurements over a seven-day period should be used to capture important fluctuations of health states (9). Mitchell et al. highlighted among 30 patients requiring kidney care in individual semi-structured interviews with the “think-aloud” technique that when patients were asked to complete the EQ-5D-5L, more than one out of ten patients made errors and struggled to complete the questionnaire (10). This was mainly due to patients not understanding the instructions for the time window and the assessment phrase “your health today” of the EQ-5D-5L.

Thus, the lack of understanding of the questionnaire and its recall period might influence the assessment of the patient's actual HRQoL status, especially in those experiencing recurrent health fluctuations (11). Therefore, health state fluctuations could also unconsciously be included in the rating of the patient's present self-reported HRQoL or by proxy-reports by caregivers. Based on these considerations, the recall period of 1 day (today's health) might not capture recurrent health fluctuations. Even “today” may cause ambiguity. For example, “today” at 10 AM might differ from “today” at 10 PM for a patient's actual self-rated health. A longer recall period, especially for those HRQoL dimensions that are likely affected by health fluctuations, could have the potential to better capture health state fluctuations, resulting in a lover variability and higher reliability of the instruments values. However, evidence suggesting this hypothesis is lacking. Therefore, it is vital to understand health state fluctuation, the affected dimensions, and if recurrent health state fluctuations were considered in the assessment of today's health.

### Objective

This study aims to (i) quantify the occurrence, frequency and intensity of health state fluctuations and underlying health events over 14 days, (ii) identify which HRQoL dimensions may be affected by these fluctuations, and (iii) evaluate if past fluctuations are considered in the assessment of health today using the EQ-5D-5L. Furthermore, if the latter is true, this study aims (iv) to determine which recall period would be more appropriate to capture health state fluctuations in dementia.

## Methods and analysis

### Study design and setting

To understand and explore the impact of health state fluctuations in dementia on the EQ-5D-5L assessment of today's health, this study will be carried out as an explorative, single-group, observational study that combines quantitative and qualitative (mixed) methods over the following four main study phases (see Table 1): (I) Quantitative baseline assessment (socio-demographics and clinical data) from both PlwD and family caregivers *via* interview at patients and caregivers home, (II) daily completion of the paper-pencil diary by the caregiver over 14 days, and (III) quantitative follow-up assessment *via* interview and (IV) qualitative semi-structured interviews at patients and caregivers homes after the 14 day assessment period.

**Table 1:** Questions of the semi-structured interview.

**#**	**Question**
1	“Were you able to complete the diary every day?”.
2	“How easy or difficult was it for you to answer the diary? Why?”
3	“Were there any fluctuations in your relative's condition? What were the fluctuations?”
4	“Were the last 2 weeks typical of his/her normal situation?”
5	“What events did you note positively in the diary? What events did you note negatively in the diary? Why?”
6	“Are the events frequent? Can you describe how often they occur?”
7	“How exactly is your relative's condition affected? What areas of health were frequently affected?”
8	“Can you describe how important this area is to your relative's health?”
9	“Do you think your relative is aware of these fluctuations?”
10	“Now that we have discussed the diary entries, I want to talk about the 5-question questionnaire (EQ-5D-5L) you completed. Is the questionnaire a good reflection of the state of your health?”
11	“How easy or difficult was it for you to answer the questionnaire?”
12	“Are some questions more difficult than others? If so, why?”
13	“In the questionnaire, you are asked to describe your relative's CURRENT state of health. On all 3 days, did you only consider your relative's current state of health? Or did you include a longer time (yesterday or last week) when answering the questionnaire?”
14	“Thinking about the last 14 days, do you think your relative's health today is a good reference for their general health?”
15	“Do you have a suggestion for an alternative time of demand?”
16	“How often do you think this questionnaire (EQ-5D-5L) should be administered to better represent your relative's health?”
17	“Do you think your relative would have answered these questions differently? If yes, why?”

After an initial socio-demographic and clinical baseline assessments of PlwD and their caregivers *via* interview at home, the caregiver will be instructed to complete a daily health diary (paper-pencil) for 14 days, documenting the PlwD's health state fluctuations, their intensity, HRQoL domains affected, and health events that could have caused the health fluctuation. HRQoL will be assessed *via* an interview using the EQ-5D-5L as self- and proxy-rating (proxy-proxy) at baseline, day seven and 14. Subsequently, the caregiver will be interviewed about the documented health state fluctuation, whether these fluctuations were considered in the assessment of the patient's today's health using the EQ-5D-5L, and (if the latter is true) what recall period would be more appropriate to capture health fluctuations in dementia.

### Participant recruitment and eligibility criteria

PlwD and their informal caregiver will be recruited in the primary care setting through specifically qualified study nurses who work with general practitioners (GP), neurologists, psychiatrists and memory clinics who are in ongoing collaborations and studies with the German Center for Neurodegenerative Diseases (DZNE) at the site Rostock/Greifswald in Mecklenburg-Western Pomerania, a predominantly rural state in the north-eastern part of Germany. Study nurses will act as gatekeepers in this study as they may be perceived as trustworthy by participants due to their previous contact in other studies. Based on the means of recruitment, participants could have been included in other recently completed studies (ClinicalTrials.gov identifiers: NCT04741932, https://clinicaltrials.gov/ct2/show/NCT01401582, NCT03359408, NCT04037501; German Clinical Trials Register Reference No: DRKS00025074, DRKS00013555). After completing these studies, the study nurse checks the patient's and caregiver's eligibility criteria for this study, informs the dyad about the new study if they are eligible, and asks them to participate. If they were willing to participate again, they would be asked to provide written informed consent (IC) and be introduced to the study procedures. If this recruitment path does not yield sufficient participants, we plan to recruit from external settings, such as memory clinics. In some cases, informed consent might require a legal representative when informed consent cannot be acquired from the person with dementia due to severe cognitive impairment.

PlwD should meet the following inclusion criteria: positively screened for dementia through the DemTect procedure (DemTect ≤ 8) (12) or formally diagnosed with dementia by a treating GP, neurologist or psychiatrist. Eligibility criteria for caregivers are as follows: living in the same household or being in contact at least once a day (seeing the patient in person) with the PlwD.

**Figure 1:**
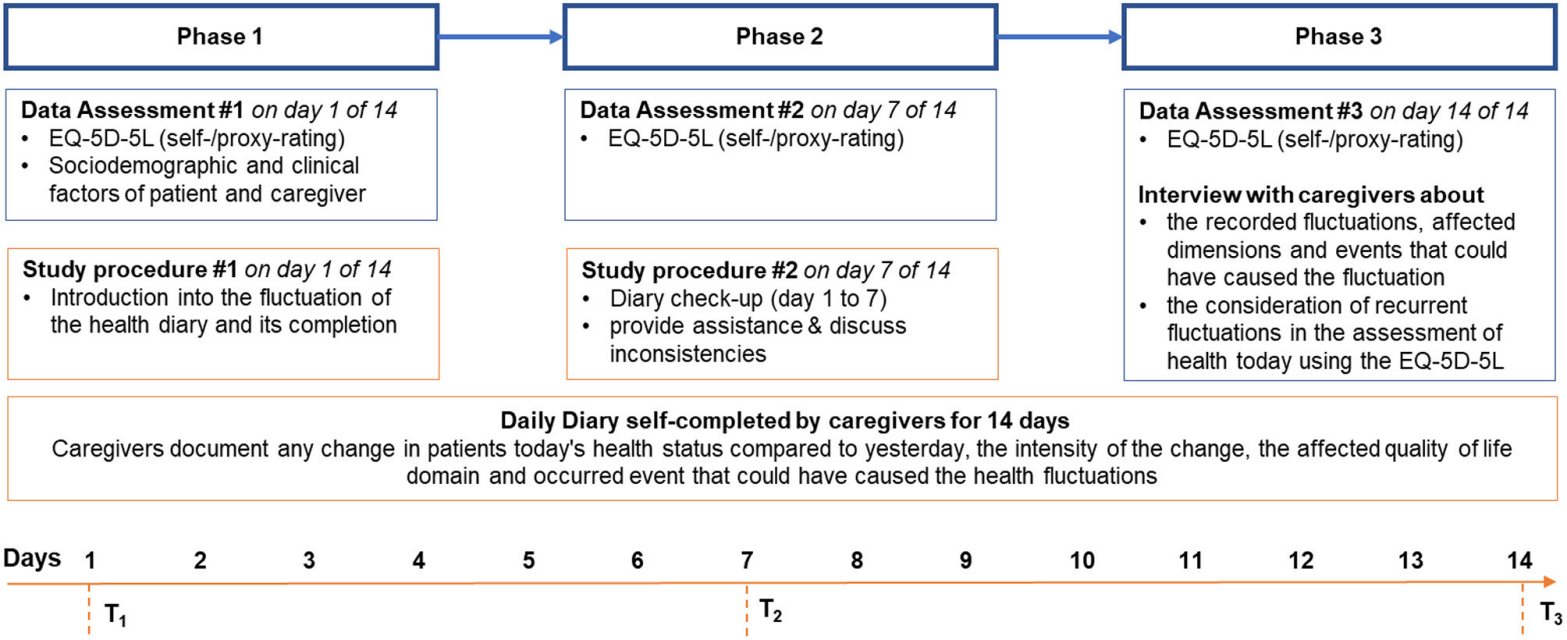
Study procedures.

Ethical approval has been obtained from the Local Ethical Committee at the University Medicine Greifswald (Registry number BB128/21 and BB 128/21a). The overall study design is illustrated in Figure 1.

### Sample size

The sample size in qualitative research is driven by data saturation. However, there are no standard criteria for calculating the sample size for this type of study. Blome et al. (9) set their sample size in the process guided by information power. We assume the sample size should be large enough to reveal at least *n* = 20 patients with health state fluctuations. Sun et al. (2) examined a sample size of *n* = 55 patients and found significant associations regarding fluctuations in HRQoL. However, the percentage of patients with dementia reporting health state fluctuations within 14 days is currently unknown. We, therefore, hypothesize that a sample size of *n* = 50 patient and caregiver dyads would be large enough to bear HRQoL outcomes (see health fluctuation outcomes below) and achieve the aims of this study.

### Data collection

At baseline, day seven and day 14, the EQ-5D-5L (13, 14) will be administered *via* standardized face-to-face interviews by dementia-specific qualified nurses at patients' or caregivers' homes. Both patients (self-rating) and caregivers (proxy-rating) will be asked to complete the EQ-5D-5L (in separate rooms). Also, patients' socio-demographic (age, sex, living situation) and the following clinical data will be assessed at baseline: cognitive impairment according to the Mini-Mental State Examination (MMSE) (15, 16), depression according to the Geriatric Depression Scale (17), perceived social support according to the F-SozU (18), deficits in daily living activities according to the Bayer Activities of Daily Living Scale (19), and comorbidity according to the Charlson Comorbidity Index (20, 21). A brief description of the assessed clinical outcome measures is demonstrated in the section “measures.”

**Figure 2:**
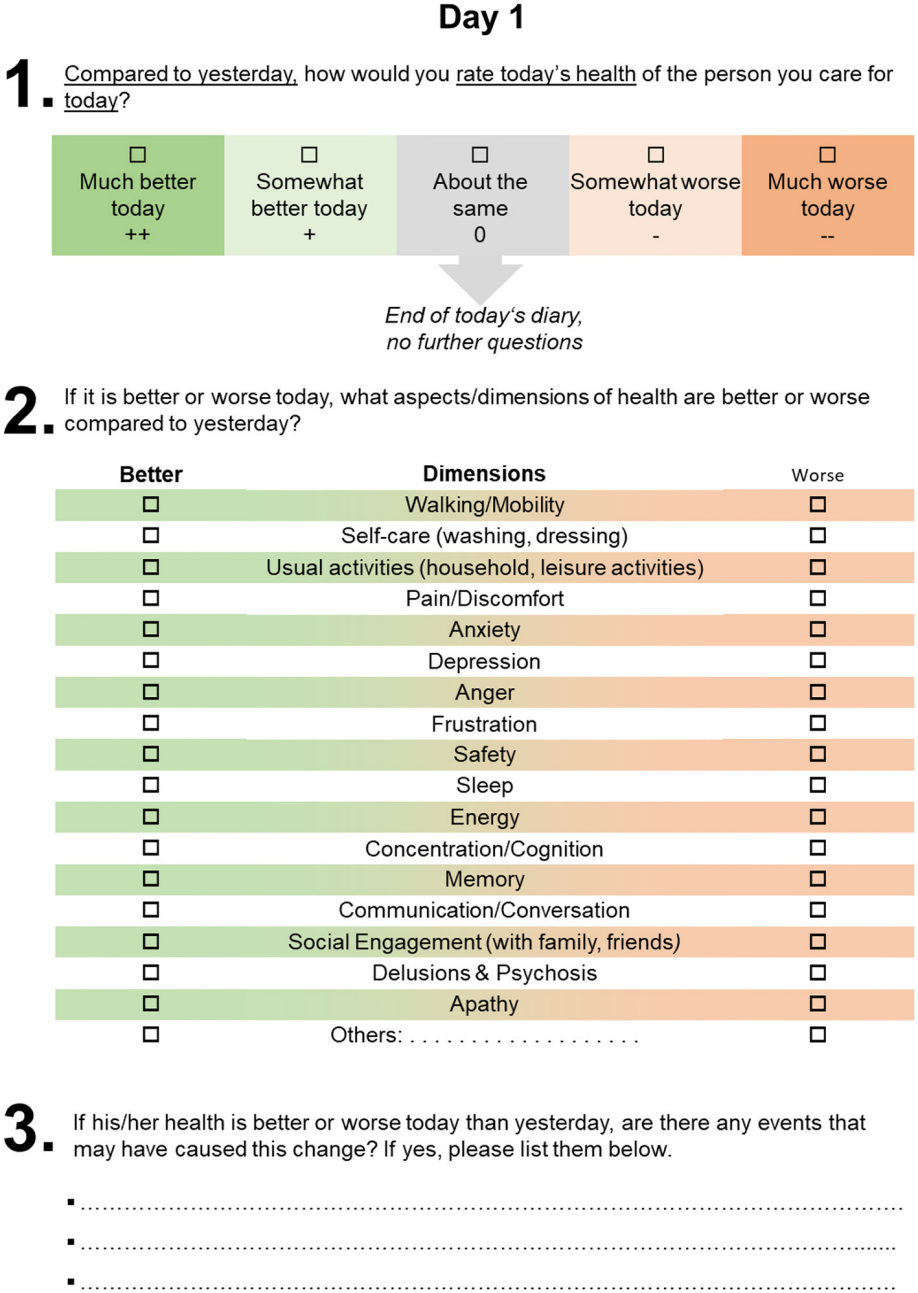
Health fluctuation diary (exemplary illustration for day 1).

After completing the baseline assessment, caregivers will daily self-complete a fluctuation diary for 14 days, documenting the frequency and intensity of health state fluctuations, affected dimensions, and underlying health events (see Figure 2). After completing the fluctuation diary, the diary data will be collected, and the study nurse will conduct a semi-structured interview on day 14 about the documented recurrent health state fluctuations, the possible consideration of these fluctuations in the assessment of health today using the EQ-5D-5L, and appropriate recall periods to capture recurrent fluctuations of HRQoL more accurately in dementia. The questions of the semi-structured interview are displayed in Table 1. Furthermore, guidelines will be drafted, and the nurses will be trained to ensure the replicability of interviews and promote data quality.

On day seven of the self-completion of the 14-day diary, the nurse will visit the caregiver at their home and will glance over the caregiver's documentation from day 1 to day 7. The study nurse will provide further assistance, discuss inconsistencies in the documented answers and be available for any questions about completing the diary. This could avoid entry errors and missings and, therefore, promotes the retention of protocols. Furthermore, instructions will be provided on how to fill out the diary.

As an incentive for complete follow-up, the patient and caregiver receive a lump sum payment upon completion of the 14-day assessment period.

Before the study roll-out, a pilot study will be implemented for *n* = 5 patients, testing the acceptability, reliability and validity of the used measures. Data collection will take place between October 2021 and December 2022.

### Measures

#### Health-related quality of life and clinical measures

The EQ-5D-5L (13) is one of the most used and well-validated generic patient-reported outcome measures that assess patients' HRQoL today. The EQ-5D-5L comprises both the EQ visual analog scale (VAS), ranging from 0 (worst imaginable health) to 100 (best imaginable health) and a descriptive system comprising five dimensions, namely, mobility, self-care, usual activities, pain/discomfort and anxiety/depression (13). Each dimension has five levels varying from no problems to extreme problems. Each level corresponds to a 1-digit number that expresses the level selected for that dimension ranging from 1 (no problem) to 5 (severe problem), with higher numbers indicating more severe problems (13). These values will be converted to a health utility index using a country-specific value set (14).

The MMSE is the most common tool for assessing the severity of cognitive impairment, categorizing participants into one of four groups of cognitive impairment: without (MMSE value ≥ 27), mild (MMSE values: 20–26), moderate (MMSE values: 10–19), and severe (MMSE values: 0–9) (15, 16). The GDS is a useful screening tool in the clinical setting to facilitate the assessment of depression in older adults, consisting of 15 items. Scores under six are considered normal, depending on age, education, and complaints, and scores above five indicate a hint of depression (17). The F-SozU is a measure of perceived and anticipated social support using 14 items that are rated on a 5-point Likert scale (1 = “does not apply at all” to 5 = “totally applies”) (18). The B-ADL assesses patients' deficits in the performance of everyday activities, comprising 25 items (19). The Charlson Comorbidity Index is the most commonly used comorbidity score to measure the burden of diseases and categorize patients comorbidities based on the International Classification of Diseases (ICD) diagnosis. A weighted sum score could be derived through the absence and presence of different comorbid conditions, with higher scores indicating higher comorbidity (20, 21).

#### Outcome measures to assess health fluctuations

The primary outcomes of this study are (i) the occurrence, frequency and intensity of health state fluctuations documented in the 14-day diary and by using the change in health states from day 1 to day 7 and day 7 to day 14 using the EQ-5D-5L index, the EQ-5D dimensions and the EQ-VAS, (ii) affected HRQoL dimensions, (iii) the underlying health events over time that could have affected patients' health, and (iv) the incorporation of recurrent health state fluctuations in the assessment of health today using the EQ-5D-5L.

i. To assess the frequency and intensity of the health state fluctuation, caregivers document if patients' today's health is much (+2) or somewhat better today (+1), about the same (±0) today, or somewhat (−1) or much (−2) worse today compared to the day before. Also, a repeated administration of the EQ-5D-5L will also assess the health state fluctuation as a self- and proxy-rating at baseline (before starting the diary), seven days after starting the diary, and on the last day of the diary (day 14). The change in the EQ-5D-5L utilities, EQ-5D-5L level scores and level sum scores (LLS), and EQ-VAS scores from day 1 to day 7 and day 7 to day 14 can be used to assess the variability and reliability of each measure.
ii. To assign health state fluctuations to specific health dimensions, we will use the health dimension of the diary and the assessment of EQ-5D-5L dimensions at baseline, seven, and 14 days. The caregiver will be instructed to document in the diary which dimensions could be assigned to the fluctuation of today's health state compared to the day before. We will use dimensions of the EQ-5D-5L and two other patient-reported outcomes measures (PROMs), the EQ Health and Wellbeing instrument (EQ-HWB) (22) and the dementia-specific Quality of Life–Alzheimer's Disease (QoL-AD) (23). The EQ-HWB has been designed as a standardized measure of aspects of health and well-being and has 25 items (24). In comparison, the QoL-AD is a widely used measure of the quality of life in dementia diseases that contains 13 items evaluating the domains of interpersonal relationships, financial difficulties, physical condition, memory, mood and overall health. Furthermore, caregivers could specify any other affected dimensions not listed in the diary. All dimensions are shown in Figure 2.
iii. Caregivers will also be asked to document any health events that could have caused the health state fluctuations.
iv. To evaluate the consideration of past health state fluctuations in the assessment of today's health, the EQ-5D-5L will be administered on day 14. Subsequently, the caregivers will be interviewed and asked whether or not recurrent health state fluctuations (if applicable) were considered in the assessment of health today using the EQ-5D-5L administered minutes before starting the interview. Furthermore, the caregiver will be asked to state what recall period would be more appropriate to assess HRQoL in people living with dementia more accurately.

### Data management

Data management will be conducted according to the guidelines and recommendations for ensuring Good Epidemiological Practice (GEP) (24). Study nurses will collect the socio-demographic and clinical data and the EQ-5D-5L using paper-pencil questionnaires during face-to-face visits with the patients and the caregivers. The diary is also on a paper-pencil basis but will be self-completed by the caregivers and collected by the nurse after completion. A medical documentary transfers all data in electronic Case Report Forms (eCRFs), including automatic plausibility and completeness checks. Physical documents will be stored in a protected place and destroyed after data transfer at the end of the data collection period. The client-server stores all data in the project data management system (25).

### Statistical analyses

#### Quantitative data preparation and analyses

Patient and caregiver characteristics will be demonstrated descriptively. Descriptive statistics will also describe health state fluctuations' occurrence, frequency, and intensity. The occurrence will be operationalized dichotomously: no fluctuations reported vs. at least one fluctuation reported. The frequency will be quantified as the number of fluctuations within 14 days. The intensity will be operationalized as follows: much worse or better = ±2; somewhat worse or better = ±1; about the same = ±0) of the reported health events. We will demonstrate the intensity of the health state fluctuations as a mean change per day with standard deviation.

Furthermore, we will use exploratory data analysis of the EQ-5D-5L data by reporting the number and percentage of patients reporting each level on each dimension of the EQ-5D-5L using simple descriptive statistics (26). The EQ-5D-5L profile and EQ-VAS scores will also be demonstrated using descriptive statistics. Also, change in EQ-5D-5L index, EQ-VAS and LLS will be demonstrated descriptively [mean value of change, Intra Class Correlation (ICC)] over the fluctuation intensity. Furthermore, cluster analyses will be used to identify frequent health events or a combination of events.

To assess the association between occurrence, frequency and intensity of the reported health fluctuation and (i) the differences between the baseline, 7-day and 14-days follow-up EQ-5D-5L profile, health utility and EQ-VAS as well as the (ii) reporting of consideration of these health state fluctuations in the assessment of health today in the EQ-5D-5L, we will use appropriate statistics such as cross tables with ANOVA, *t*-tests and multiple logistic regression models. According to the latter one, multivariate regression models will include the adherence to the recall period (dichotomous: yes, no) as a dependent variable and the fluctuation intensity (low, moderate, high) and further socio-demographic and clinical variables of the patient as an independent variable. We hypothesize that frequent and more intensive health fluctuations reported in the diaries will cause higher variability of the EQ-5D-5L index between baseline and seven or 14 days later and will have higher odds for consideration of these fluctuations in the assessment of HRQoL today (non-adherence to the recall period of today). Additionally, we will use descriptive statistics to identify specific health events most likely affecting the incorporation into the health assessment today. Statistical analysis will be carried out in SAS (version 9.4 M7).

#### Qualitative data preparation and analysis

The qualitative interviews will be transcribed, coded using NVivo (version 12, Germany) and analyzed thematically (27). A thematic analysis consists of the following stages: (i) familiarization, (ii) identifying a thematic framework, (iii) indexing, (iv) charting, and (v) mapping and interpretation. This approach is generally considered more appropriate when the data collection tends to be more structured and there are pre-determined issues to explore. As such, a deductive approach will be used with a pre-determined framework around the following themes: occurrence and frequency of health fluctuations, the intensity of fluctuation, affected dimensions, and recall period. To increase validity of qualitative research results, two independent researchers will analyze the interviews separately, and checkups, will be made in the beginning to test for consistency and widely disparate coding.

Results of the thematic analysis of the semi-structured interviews will be compared with the results of the diaries and the EQ-5D-5L assessment to demonstrate how often and intensively health fluctuates in our sample of 50 patients with dementia, what dimensions were affected by these fluctuations, whether documented past events and health fluctuations were considered in the assessment of health today using the EQ-5D-5L, and what recall period would be more appropriate from the perspective of the caregivers to assess health status in such fluctuating conditions.

## Discussion

To the best of our knowledge, this will be the first study evaluating the health state fluctuations in dementia diseases using a daily diary to document the change in today's health compared to the day before over 14 days. This study may reveal important insights into the frequency and intensity of health state fluctuations in dementia, the affected dimensions and underlying health events. Analogous to the findings of Sun et al. (2), we assume that most patients will be affected by health state fluctuations and that these fluctuations impact different health dimensions. Furthermore, we expect to see that health state fluctuations are associated with a higher variation in the assessed HRQoL using the EQ-5D-5L. Therefore, the results of this study may reveal whether and how the five EQ-5D-5L dimensions capture fluctuations in health status in PlwD and whether individuals adhere to the recall period by assessing the extent to which they considered past events and health state fluctuation within their ratings for the health today. If the latter is the case, the interviews will provide information about a more appropriate recall period that captures relevant health state fluctuations and enables a more comparative and in-depth assessment of health status. Like Blome et al. (9), it might be more appropriate to test for EQ-5D-5L several times within a recall period to retain more reliable results.

The information generated from this mixed-methods study can also be vital to inform future research to test modified recall periods of the EQ-5D-5L in PlwD or other fluctuating cyclical health state conditions. The results of this study could make a highly relevant first contribution to more accurate measurements and, thus, give a better understanding of the HRQoL of PlwD. A better understanding of the influences of health state fluctuations on the EQ-5D-5L will make generic PROMs in cyclical health conditions more reliable and generalizable. It will propose a hypothesis on better recall and testing periods for the EQ-5D-5L and other PROMS. Furthermore, the additional information on the presence of fluctuations, the influence of the one-day capture of the EQ-5D-5L and potential changes in this measure necessary to better integrate such information may help to increase the treatment and counseling of patients and their caregivers.

### Limitations

Due to the COVID-19 situation in Germany, it has to be noticed that recruitment took place under challenging and changing conditions. Especially aged adults are affected by the COVID-19 diseases and the imposed measures to protect against the spread of the disease (28). These circumstances affect the data assessments at patients' and caregivers' homes and might influence recruitment. To accurately monitor health fluctuations and discover the influences of affected health dimensions, this study relies on completing the 14-day diary. Despite checkups on the diary on day 7 and day 14, caregivers might still miss reporting health state fluctuations on days that they are unsupervised and thus might underestimate the influence of health state fluctuations.

Additionally, there are significant differences in the perception of health status between the caregiver and the patient (self and proxy). Findings reported by the caregiver might pronounce the caregiver's perspective on health state fluctuations in PlwD (29, 30). We will address these problems by triangulating our quantitative findings with the interview findings and focusing on the patient's perspective in multivariable regression models.

Finally, the scope of this study will be limited to the federal state of Mecklenburg-Vorpommern. However, previous studies in dementia care research in this state have shown that findings are generalizable to other results nationwide.

## Trial status

Protocol Version 08-30-2022: Recruitment for the pilot phase began on the 15^th^ of December 2021 and will probably be completed by the end of January 2022. The roll-out of the final data assessment is planned for February 2022 and is estimated to be finalized by December 2023.

## Ethics statement

The studies involving human participants were reviewed and approved by University Medicine Greifswald (Registry numbers BB128/21 and BB128/21a). The patients/participants provided their written informed consent to participate in this study.

## Author contributions

The trial was conceptualized by BM, FX, LE, and TK. BM has contributed substantially to the concept of the study and to the draft of the study protocol and will supervise the quantitative analyses. NW is responsible for study processes and trial coordination. LE will be responsible for the qualitative analyses. NW and BM have drafted the manuscript. All authors provided input to, reviewed the manuscript, and approved the final manuscript.
